# Patterns of regional lymph node failure of locally advanced hypopharyngeal squamous cell carcinoma after first-line treatment with surgery and/or intensity-modulated radiotherapy

**DOI:** 10.1186/s12885-020-06793-6

**Published:** 2020-04-06

**Authors:** Dongqing Wang, Shui Yu, Limin Zhai, Jin Xu, Baosheng Li

**Affiliations:** 1 Tianjin Medical University, Tianjin, 300070 P.R. China; 2 Department of Radiation Oncology, Shandong Cancer Hospital and Institute, Shandong First Medical University and Shandong Academy of Medical Sciences, Jinan, 250117 P.R. China

**Keywords:** Hypopharyngeal squamous cell carcinoma, Surgery, Radiotherapy, Chemotherapy, Failure pattern

## Abstract

**Background:**

To identify the spatial patterns of regional lymph node failure of locally advanced hypopharyngeal squamous cell carcinoma (SCC) after first-line treatment with surgery and/or intensity-modulated radiotherapy (IMRT).

**Methods:**

We retrospectively obtained the clinicopathological characters of 123 hypopharyngeal SCC patients, and investigated the patterns of regional lymph node failure. Univariate and multivariate logistic regression were used to determine the risk factors of regional lymph node failure.

**Results:**

Forty patients (32.5% of total patients) were suffered regional lymph node failure. In these patients, the ipsilateral neck level II nodal failure account for 55.0% (22/40) followed by level III 30.0% (12/40), level VIb 15.0% (6/40), level VII 15.0% (6/40), and level IV 5.0% (2/40). In addition, 17.5% (7/40) patients suffered contralateral neck level II nodal failure and 7.5% (3/40) patients suffered level III nodal failure. The common failure levels were the II (7/46, 15.2%), III (4/46, 8.7%), VIb (4/46, 8.7%), and VII (5/46, 10.9%) for treatment by surgery. The lymph node recurrence and persistent disease at levels II (19/77, 24.7%) and III (10/77, 13.0%) remained the major cause of failure following curative intent of IMRT. The postoperative radiation significantly decreased the risk of regional lymph node failure (OR = 0.082, 95% CI: 0.007–1.000, *P* = 0.049); and the radiologic extranodal extension significantly increased the risk of regional lymph node failure (OR = 11.07, 95% CI: 2.870–42.69, *P* < 0.001).

**Conclusions:**

Whatever the treatment modality, the lymph node failure at level II and III was the most popular pattern for hypopharyngeal SCC. Moreover, for patients who underwent surgery, the nodal failure at level VIb and VII was frequent. Thus, postoperative radiation of level VIb and VII may give rise to benefit to locally advanced hypopharyngeal SCC patients.

## Background

Squamous cell carcinoma (SCC) of the hypopharynx is relatively rare and accounts for 3 to 7% of all head and neck cancers [1–3]. Notably, the hypopharyngeal SCC is a lethal disease and the 10-year overall survival is only 13.8% [4, 5]. The poor prognosis of hypopharyngeal SCC may result from the facts that early stage of hypopharyngeal SCC often fails to cause any signs or symptoms, and this delays the diagnosis of hypopharyngeal carcinoma [1–3]. Currently, the standard treatment for locally advanced hypopharyngeal SCC is multimodality treatment, including induction chemotherapy, partial or total laryngopharyngectomy with lymph node dissection, and postoperative radiation or chemoradiation as dictated by pathologic risk features, such as, positive margins or extranodal extension (ENE) [6, 7]. As for advanced unresectable tumors, such as stage IVb diseases, and for patients requiring organ preservation, concurrent radiotherapy (RT) and high-dose cisplatin is recommended treatment schedule in national comprehensive cancer network (NCCN) guideline for cancer of hypopharynx [7].

The intensity-modulated radiotherapy (IMRT) plays an important role as an adjunct to surgery or concurrent with chemotherapy. Accurate target volume delineation is critical to achieve favourable clinical outcomes. Recently, Biau et al. [8] updated the international consensus guidelines for the delineation of the neck node levels of head and neck cancers. However, there is still no consensus on the extent to which prophylactic treatment regional nodal basin needs to be included in adjuvant IMRT. Moreover, the pattern of the lymph node failure is still unclear in hypopharyngeal SCC patients.

In present study, we reported the follow-up results of frequency and distribution of lymph node failure at each nodal level for 123 patients with locally advanced hypopharyngeal SCC undergoing first-line treatment with surgery and/or IMRT.

## Methods

### Population

**Fig. 1:**
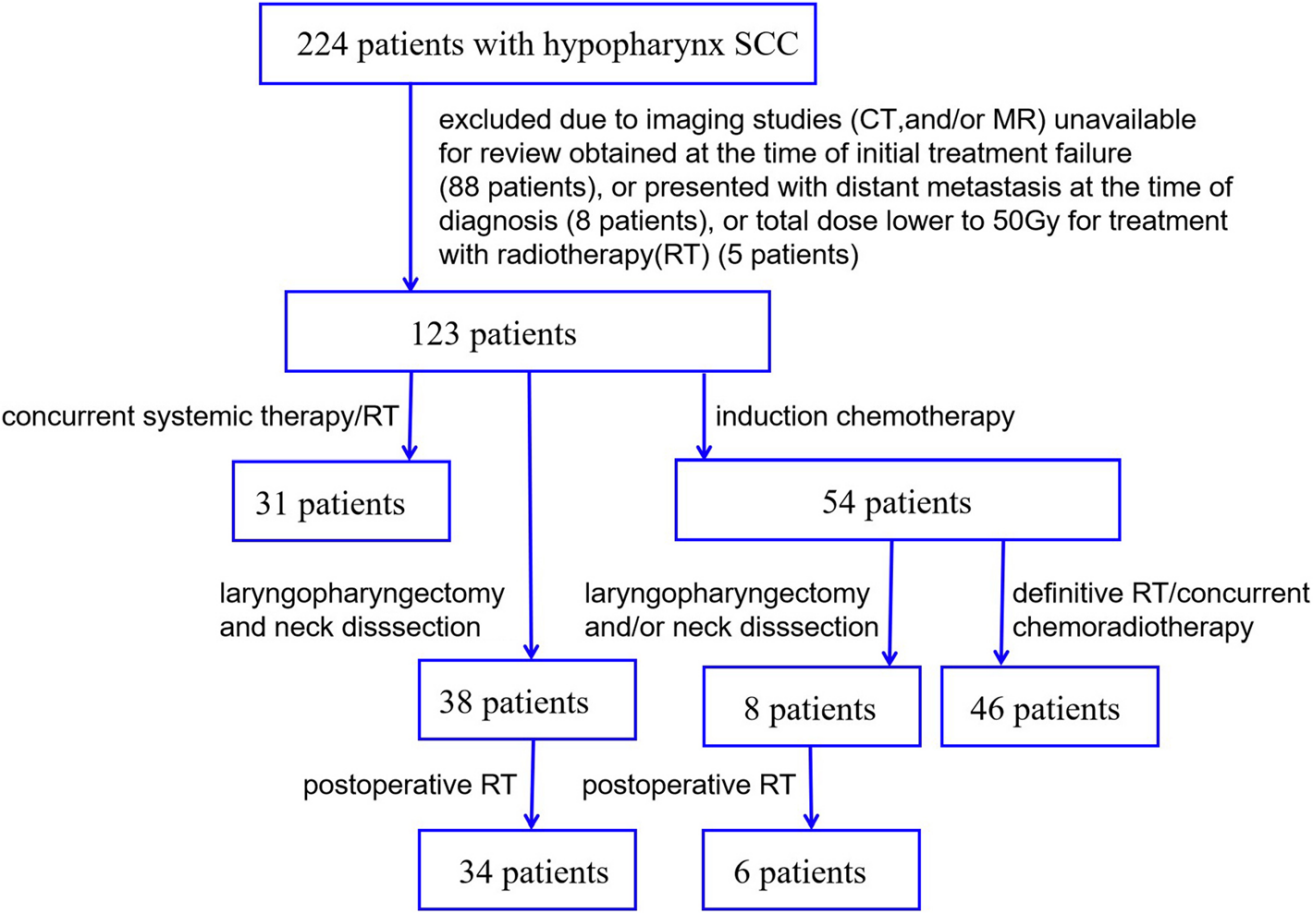
The flow diagram for the inclusion

Patients who were diagnosed as hypopharyngeal SCC and confirmed by pathology at the Shandong Cancer Hospital from January 2012 to November 2018 were retrospectively reviewed. The inclusion criteria for the present study were: (1) Clinical or pathological TNM stage II–IV_b_ according to AJCC 7th TNM classification without distant metastasis, and (2) patients undergoing radical surgery or IMRT. As indicated in Fig. 1, we excluded the patients (1) who presented with organ metastasis; (2) the radiation dose lower to 50Gy; and (3) imaging studies unavailable for review at the time of initial treatment failure. The protocol of this study was approved by the Institutional Review Board of the Shandong Cancer Hospital.

### Surgery treatment

Totally, 20 patients received total pharyngolaryngectomy with unilateral neck dissection for 14 patients, bilateral neck dissection for 6 patients. In addition, 20 patients received partial pharyngolaryngectomy with unilateral neck dissection for 15 patients, bilateral neck dissection for 5 patients. Notably, 6 patients were not treated by laryngopharyngectomy after induction chemotherapy, and only underwent the isolated unilateral neck dissection and postoperative RT. One patient could not be treated by laryngopharyngectomy for his poor cardiopulmonary function. One patient with stage T_1_N_2b_M_0_ acquired disease progression for nodal disease, and complete response for primary tumor after induction chemotherapy. Four patients would like to preserve the larynx and only removed the lymph node. The type of performed neck dissection was selective dissection. Generally, neck dissection involved levels II, III, IV and V. Level I and VIb were removed in partial patients according to tumor site, T stage, and lymph node metastasis on preoperative imaging.

### Radiotherapy treatment

A total 117 (95.1%) patients received IMRT during the whole treatment procedure. Of these patients, 77 received chemoradiotherapy or definitive radiotherapy alone, 40 received postoperative radiotherapy. Irradiation is applied as a step and shoot IMRT technique using 6-MV X ray in daily fractions of 1.8–2.2 Gy from Monday to Friday. For definitive radiation therapy, the gross tumor volume (GTV) encompass the primary tumor and involved nodes. The clinical target volume (CTV) contains the GTV and areas of potential microscopic spread as well as the lymph node areas for elective lymph node irradiation. The planning target volume (PTV) accounts for set-up variations by a margin of 0.3 cm. Generally, we prescribe median dose 70Gy to gross tumor, 60Gy to high-risk subclinical regions, 50Gy to low-risk subclinical regions. As for postoperative radiation therapy, high-risk regions (CTV_high_) were given 60–66 Gy, and the low-risk regions (CTV_low_) 50 Gy in daily fractions of 1.8–2.0 Gy. The extension of the CTVs was defining by radiation oncologists taking into account of clinical factors including TNM stage, number and distribution of positive lymph nodes, size of metastatic lymph nodes, extension of primary tumor beyond the midline, pathological resection status, and existence of extra-capsular spread of nodal disease.

### Systemic therapy

Twenty-eight (22.8%) patients received concurrent chemoradiotherapy, of 25 patients received cisplatin 75 mg/m^2^ as concurrent agents, 2 received nimotuzumab and 1 received cetuximab. Fifty-four patients (43.9%) received induction chemotherapy. The induction chemotherapy regimens were as follows: (1) cisplatin 75 mg/m^2^ plus 5-fluorouracil (5-FU) 750–1000 mg/m^2^/d from days 1–4 infusion, (2) docetaxel 75 mg/m^2^ on day 1 plus cisplatin 75 mg/m^2^. Three patients received cisplatin 75 mg/m^2^ plus docetaxel 75 mg/m^2^ plus 5-FU 500 mg/m^2^/d from days 1–4 infusion. Induction chemotherapy was administered in 1–3 cycles every 3 weeks.

### Regional lymph node failure

The location of lymph nodes metastasis was divided into several levels in the present study according to the DAHANCA, EORTC, HKNPCSG, NCIC CTG, NCRI, RTOG, TROG consensus guidelines for the delineation of the neck node levels [9]: Ia, submental group; Ib, submandibular group; II, upper jugular group, and level II is further subdivided into level IIa and level IIb by the posterior edge of the internal jugular vein; III, middle jugular group; IV, lower jugular group; V, posterior triangle group, and level V is further subdivided into levels Va (upper posterior triangle nodes) and Vb (lower posterior triangle nodes) using the caudal edge of the cricoid cartilage as an anatomic landmark; VIa, anterior jugular nodes; VIb, prelaryngeal, pretracheal, and paratracheal nodes; VII, retropharyngeal nodes.

Local recurrence was defined as recurrence at the site of the initial primary tumor, and regional failure was defined as the development of recurrence in cervical lymph nodes. Distant failure was defined as metastasis in an organ outside of the head and neck. The presence of failure was determined based on the information of a clinical evaluation, systemic radiographic imaging and biopsy, and it was evaluated by Dongqing Wang and Shui Yu.

Lymphatic metastasis intensity (LMI) was used to describe as the ratio of the number of positive lymph nodes to the number of examined lymph nodes. Lymphatic metastasis ratio (LMR) was defined as the ratio of the number of patients with positive lymph node diagnosed by contrast-enhanced CT and/or magnetic resonance imaging divided by the number of the whole population.

### Follow-up

After completion of treatment, patients were followed by every 3 months for the initial 3 years, and every 6 months after 3 years. Progression-free survival (PFS) was considered as the time period from treatment completion to the initial treatment failure.

### Statistical analysis

Statistical analysis was performed using the SPSS statistical software, version 20.0 (IBM Corporation, Armonk, NY, USA). LMI and LMR were presented as the frequencies and percentages. The mean PFS were determined by the Kaplan-Meier curve (log-rank test). The lymph node recurrence rate at respective level in patient treated with surgery, with or without postoperative RT was analyzed by Chi-square test. The univariate and multivariate logistic regression were used to determine the risk factors of lymph nodal failure. The factors included age, TNM stage, adjuvant treatment with postoperative RT and chemotherapy, and radiologic extranodal extension (rENE). *P* values of < 0.05 indicated significant difference.

## Results

### Clinicopathological characters

The clinicopathological characters were summarized in Table 1. The median age was 58 years (range, 41 to 82 years) and majority was males (95.9%). Hypopharyngeal subsite was piriform sinus in 106 cases (86.2%), posterior hypopharyngeal wall in 9 (7.3%), and retrocricoid in 8 (6.5%). According to AJCC 7th criteria, clinical or pathological staging were 7 (5.7%) for stage II, 25 (20.3%) for stage III and 91 (74.0%) for stage IV. One hundred and seventeen (95.1%) patients underwent IMRT, 40 (32.5%) postoperatively and 77 (62.6%) definitively. Forty patients (32.5%) received total or partial pharyngolaryngectomy with neck dissection, 6 received isolated unilateral neck dissection. Twenty-eight (22.8%) patients received concurrent chemoradiotherapy, and 54 (43.9%) received induction chemotherapy. In addition, we summarized the treatment schedule based on the T and N classification (Table 2).

**Table 1:** Patient, disease, and treatment characteristics

Characteristics	N (%)
Age, years	
Median (range)	58 (41–82)
Gender	
Male	118 (95.9%)
Female	5 (4.1%)
Tumor site	
Pyriform sinus	106 (86.2%)
Posterior pharyngeal wall	9 (7.3%)
Postcricoid area	8 (6.5%)
TNM stage	
II	7 (5.7%)
III	25 (20.3%)
IV_a_	83 (67.5%)
IV_b_	8 (6.5%)
Clincal T stage	
T_1_	7 (5.7%)
T_2_	19 (15.4%)
T_3_	29 (23.6%)
T_4_	28 (22.8%)
Pathological T stage	
T_1_	5 (4.1%)
T_2_	12 (9.8%)
T_3_	10 (8.1%)
T_4_	13 (10.6%)
Clincal N stage	
N_0_	12 (9.8%)
N_1_	16 (13.0%)
N_2a_	2 (1.6%)
N_2b_	25 (20.3%)
N_2c_	19 (15.4%)
N_3_	3 (2.4%)
Pathological N stage	
N_0_	6 (4.9%)
N_1_	7 (5.7%)
N_2a_	1 (0.8%)
N_2b_	26 (21.1%)
N_2c_	5 (4.1%)
N_3_	1 (0.8%)
Surgery	
Total laryngopharyngectomy and neck dissection	20 (16.3%)
Partial laryngopharyngectomy and neck dissection	20 (16.3%)
Isolated neck dissection	6 (4.9%)
Node dissection	
Ipsilateral	35 (28.5%)
Bilateral	11 (8.9%)
Intensity-modulated radiotherapy	
Postoperative	40 (32.5%)
Definitive	77 (62.6%)
Chemotherapy	
Induction	54 (43.9%)
Concurrent	28 (22.8%)

**Table 2:** Treatment schedule by clinical/pathological T and N classification

T	N	Cases	Treatment schedule (N, %)
T_1–2_	N_0_	7	S ± RT (3, 42.8%) RT (2, 28.6%) IC + RT (2, 28.6%)
T_1–2_	N_1–3_	36	IC + S + RT (2, 5.6%) IC + RT/CRT (14, 38.9%) S ± RT/CRT (14, 38.9%) RT/CRT (6, 16.6%)
T_3_	N_0–3_	39	IC + S + RT (4, 10.3%) IC + RT/CRT (16, 41.0%) S + RT/CRT (9, 23.1%) RT/CRT (10, 25.6%)
T_4_	N_0–3_	41	IC + S + RT (2, 4.9%) IC + RT/CRT (14, 34.1%) S ± RT/CRT (12, 29.3%) RT/CRT (13, 31.7%)

Note: *S* Surgery, *RT* Radiotherapy, *CRT* Chemoradiotherapy, *IC* Induction chemotherapy

### Metastasis of lymph node

Forty-six neck dissections were performed: 35 ipsi- and 11 bi-lateral, 1148 lymph nodes were analyzed. A total of 169 nodes in 40 (40/46, 86.9%) patients confirmed lymphatic metastasis, the overall LMI was 14.7% (169/1148). The LMI for ipsilateral neck was 16.4% (160/976), whereas, only 5.2% (9/172) for contralateral neck. In addition, we evaluated the LMI based on the level of lymph node. We observed that the LMI was 20.0% (14/70) for level II, 14.9% (7/47) for level III, 5.9% (3/51) for level IV, 0% (0/27) for level V, 8.0% (2/25) for level VI, and 0% (0/5) for level VII. Seventy-seven patients did not receive resection of neck, and the LMR were 66.2% (51/77) for level II, 48.1% (37/77) for level III, 13.0% (10/77) for level IV, 5.2% (4/77) for level V, 13.0% (10/77) for level VI, and 15.6% (12/77) for level VII.

### Regional lymph node failure

**Fig. 2:**
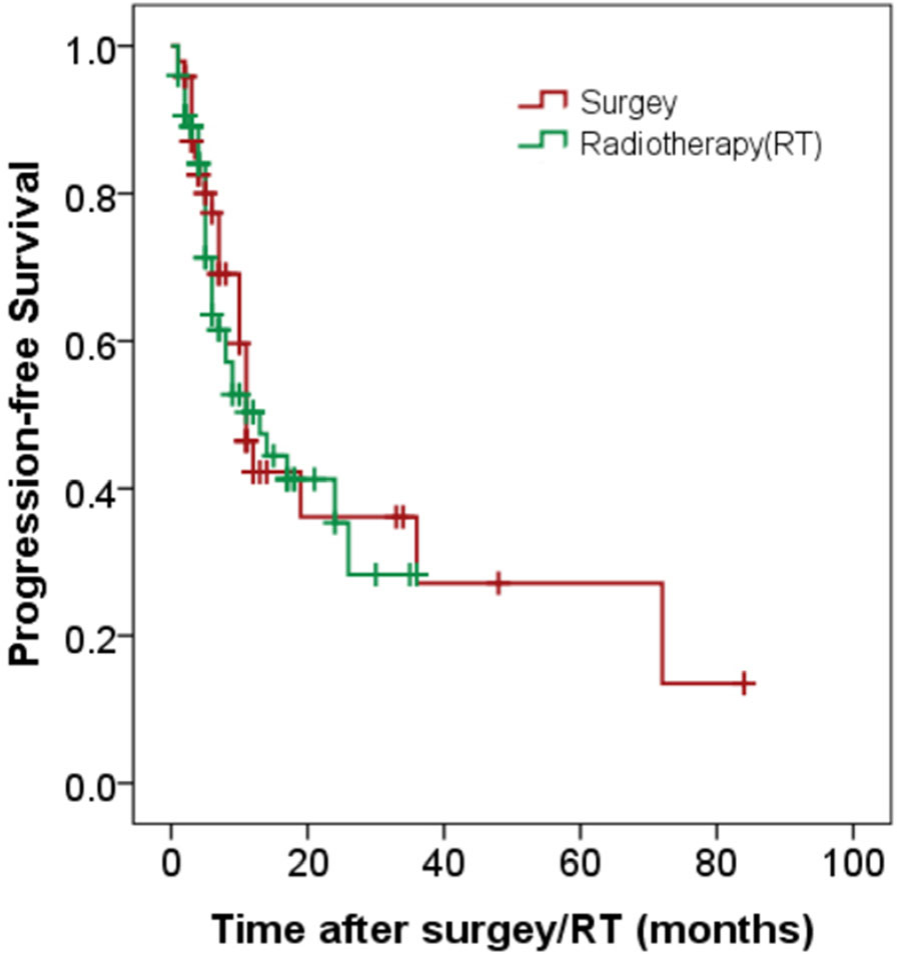
Progression-free survival of hypopharyngeal carcinoma following radical surgery and/or radiotherapy

**Fig. 3:**
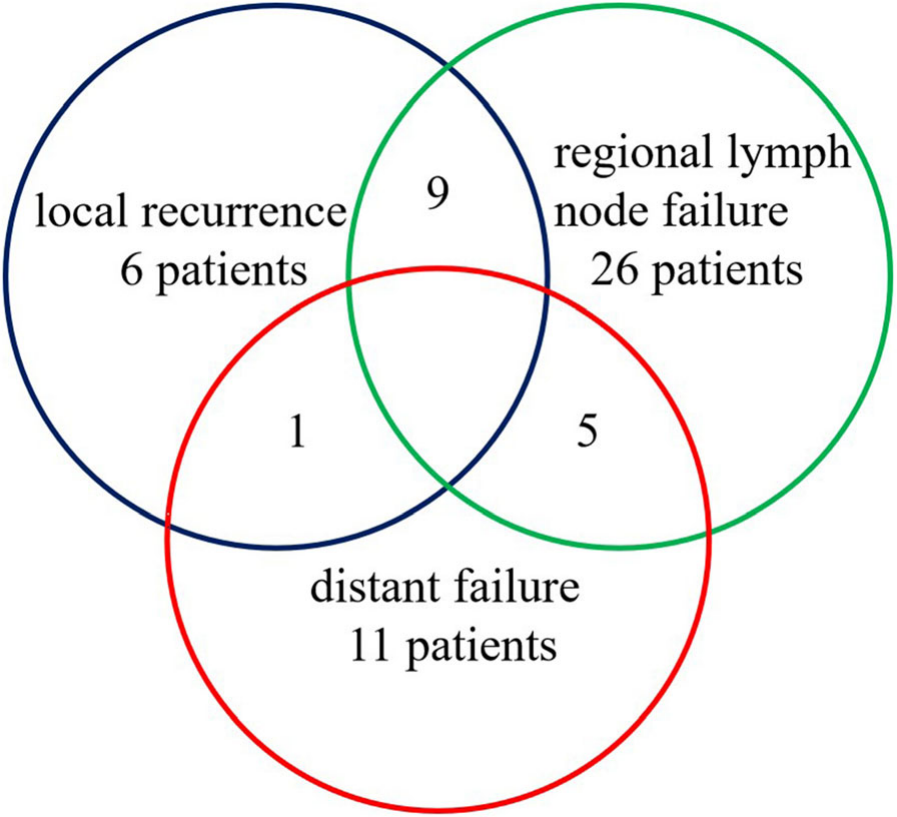
Patterns of failure of hypopharyngeal carcinoma following radical surgery and/or radiotherapy

All patients were followed up for median time 12 months (3–84 months). The median PFS rates were 13 months (95% CI 6.4–19.6 months) for surgery treatment, and 11 months (95% CI 9.1–12.9 months) for non-surgery treatment, no significant difference was observed (*P* = 0.732) (Fig. 2). For all patients, local recurrence, cervical lymph node failure, and distant metastasis accounted for 13.0% (16/123), 32.5% (40/123), and 13.8% (17/123), respectively. Of the cervical lymph node failure, 26 patients were isolated regional lymph node failure, 9 were both nodal failure and local recurrence, and 5 were both nodal failure and distant metastasis (Fig. 3). The second primary cancers were found in 19 patients (15.4%), with esophagus cancer 18 patients, and lung cancer one patient.

**Fig. 4:**
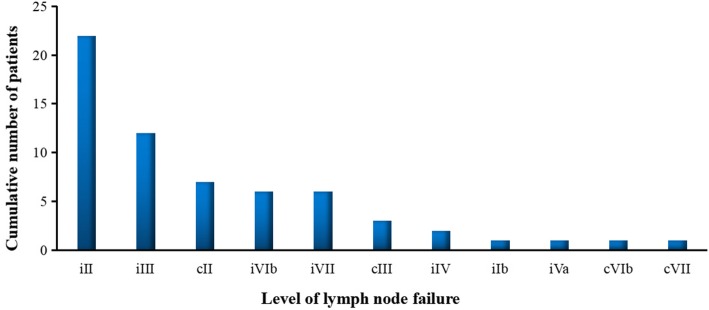
The spatial patterns of lymph node failure of hypopharyngeal carcinoma following radical surgery and/or radiotherapy

**Fig. 5:**
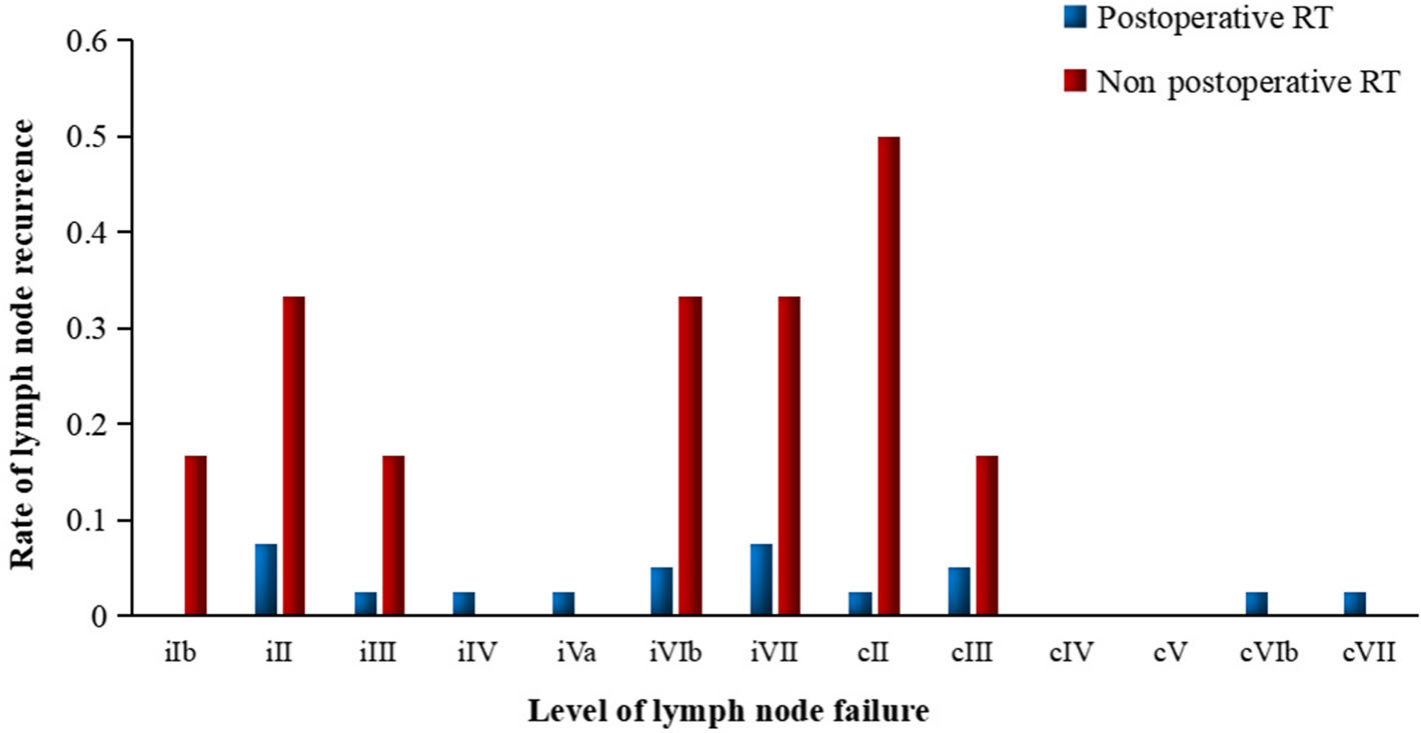
The lymph node recurrence rate at respective level for hypopharyngeal carcinoma undergoing surgery with or without postoperative radiotherapy (RT)

Of the 40 regional nodal failures, failures involved ipsilateral neck level II in 22 patients (55.0%), III in 12 patients (30.0%), IV in 2 patients (5%), VIb and VII both in 6 patients (15.0%). The nodal failures involved contralateral neck level II in 7 patients (17.5%), III in 3 patients (7.5%). Furthermore, respective one patient was found nodal failure at level Ib and Va in ipsilateral neck, and level VIb and VII in contralateral neck (Fig. 4). Notably, another one patient occurred axillary lymphatic failure accompanied by bone metastasis. For patients undergoing surgery, the most commonly failure levels were the II (7/46, 15.2%), III (4/46, 8.7%), VIb (4/46, 8.7%), and VII (5/46, 10.9%). The detailed results of lymph node recurrence at respective level was reported in Table 3 for patients undergoing surgery with or without postoperative RT. The rate of lymph node failure at levels II, III, VIb, and VII was observed higher for patients who did not receive postoperative irradiation (Fig. 5), however, probably because of small sample size (*N* = 6), borderline significant difference was observed at level VII (33.3% vs. 7.5%, *P* = 0.058, OR = 0.162, 95% CI: 0.021–0.128), and no significant difference at level III (Table 3). In contrast, for patients undergoing IMRT, the most commonly failure levels were the II (19/77, 24.7%), and III (10/77, 13.0%), then followed by VIb (2/77, 2.6%), VII (1/77, 1.3%), and IV (1/77, 1.3%).

**Table 3:** The lymph node recurrence (N, %) at respective level in patient with hypopharyngeal carcinoma treated by surgery with or without postoperative RT

Nodal level	Postoperative RT *N* = 40	Non postoperative RT *N* = 6	Total *N* = 46	*P* value	Odds ratio (95% CI)
iIb	0	1 (16.7%)	1 (2.2%)		
iII	3 (7.5%)	2 (33.3%)	5 (10.9%)	0.058	0.162 (0.021–0.128)
iIII	1 (2.5%)	1 (16.7%)	2 (4.3%)	0.113	0.128 (0.007–2.387)
iIV	1 (2.5%)	0	1 (2.2%)		
iVa	1 (2.5%)	0	1 (2.2%)		
iVIb	2 (5.0%)	2 (33.3%)	4 (8.7%)	0.022	0.105 (0.011–0.964)
iVII	3 (7.5%)	2 (33.3%)	5 (10.9%)	0.058	0.162 (0.021–0.128)
cII	1 (2.5%)	3 (50.0%)	4 (8.7%)	< 0.001	0.026 (0.002–0.328)
cIII	2 (5.0%)	1 (16.7%)	3 (6.5%)	0.280	0.263 (0.020–3.456)
cIV	0	0	0		
cV	0	0	0		
cVIb	1 (2.5%)	0	1 (2.2%)		
cVII	1 (2.5%)	0	1 (2.2%)		

Note: *i* ipsilateral neck, *c* contralateral neck, *RT* Radiotherapy

### Risk factors for lymph node failure

Table 4 showed the risk factors of lymph node failure for patients treated by surgery. The postoperative radiation strongly associated with lower risk nodal failure (OR = 0.086, 95% CI: 0.009–0.814, *P* = 0.012), and pathologic N stage had a trend towards significance on univariate analysis (OR = 0.218, 95% CI: 0.042–1.142, *P* = 0.057). In multivariate analysis, non postoperative radiation was an independent risk factor (OR = 0.082, 95% CI: 0.007–1.000, *P* = 0.049). Table 5 reported the radiologic extranodal extension (OR = 11.07, 95%: CI 2.870–42.69, *P* < 0.001) was significantly increased the lymph node recurrence and persistence for patients treated by IMRT.

**Table 4:** Univariate and multivariate analysis of lymph node failure in patient with hypopharyngeal carcinoma treated by surgery (*N* = 46)

Variable	Ipsilateral nodal failure *N* = 10	Contralateral/Bilateral nodal failure *N* = 6	Univariate Odds ratio (95% CI)	*P* value	Multivariate Odds ratio (95% CI)	*P* value
Age (years)			2.852 (0.666–12.22)	0.149	1.727 (0.348–8.563)	0.503
41–58	8	6				
58–82	2	0				
TNM stage			1.654 (1.299–2.106)	0.170	1.045 (0.508–2.151)	0.905
II	0	0				
III-IV	10	6				
Pathologic T stage			0.862 (0.256–2.894)	0.809	1.126 (0.054–23.63)	0.939
T_1–2_	4	2				
T_3–4_	6	4				
Pathologic N stage			0.218 (0.042–1.142)	0.057	0.201 (0.006–6.691)	0.370
N_0–1_	0	2				
N_2–3_	10	4				
Postoperative radiation			0.086 (0.009–0.814)	0.012	0.082 (0.007–1.000)	0.049
Yes	8	3				
No	2	3				
Chemotherapy			1.008 (0.299–3.403)	0.989	1.559 (0.311–7.813)	0.589
Yes	5	4				
No	5	2

**Table 5:** Univariate and multivariate analysis of lymph node failure in patient with hypopharyngeal carcinoma treated by intensity-modulated radiotherapy (*N* = 77)

Variable	Ipsilateral nodal failure *N* = 21	Contralateral/Bilateral nodal failure *N* = 3	Univariate Odds ratio (95% CI)	*P* value	Multivariate Odds ratio (95% CI)	*P* value
Age (years)			1.488 (0.555–3.992)	0.429	1.777 (0.545–5.797)	0.340
41–58	12	2				
58–82	9	1				
TNM stage			1.104 (1.012–1.204)	0.120	0.875 (0.141–5.420)	0.886
II	0	0				
III	5	0				
IV	16	3				
Clinical T stage			1.520 (0.548–4.215)	0.420	1.414 (0.749–2.670)	0.285
T_1_	3	1				
T_2_	5	0				
T_3_	8	2				
T_4_	5	0				
Clinical N stage			0.627 (0.222–1.772)	0.377	0.812 (0.225–2.937)	0.751
N_0_	1	0				
N_1_	5	0				
N_2_	13	3				
N_3_	2	0				
rENE			12.25 (3.353–44.75)	< 0.001	11.07 (2.870–42.69)	< 0.001
Yes	11	1				
No	10	2				
Chemotherapy			0.761 (0.200–2.894)	0.668	0.500 (0.106–2.359)	0.381
Yes	17	3				
No	4	0				

Note: *rENE* Radiologic extranodal extension

## Discussion

Our results demonstrate that 47.2% of the hypopharyngeal SCC patients were found local-regional failure and distant metastasis with median time to the initial treatment failure was 13 months (95% CI 6.4–19.6 months) for surgery, and 11 months (95% CI 9.1–12.9 months) for IMRT. The most commonly failures in hypopharyngeal SCC are mainly attributed to cervical lymph node failure, account for 32.5% of patients.

It is well know that hypopharyngeal carcinoma characterized by aggressive clinical behavior and high risk tendency to invade cervical lymph nodes. The lymph node metastasis is an important prognostic factor, therefore, control of regional metastasis is an essential part of treatment for hypopharyngeal cancer. Presently, there is no agreement on the best treatment approach for hypopharyngeal SCC. Definitive chemoradiation strategy arose from the RTOG 91–11 trial [10, 11] which demonstrated improved loco-regional control and laryngeal preservation rates has become an important approach for locally advanced hypopharyngeal cancer. By means of prophylactic neck irradiation (PNI), the incidence of nodal failure can be reduced to 4% in head and neck cancers [12]. Therefore, PNI is an important IMRT component in the treatment of hypopharyngeal cancer.

In the present study, nodal involvement mainly concerned levels II (66.2%) and III (48.1%), then followed by levels IV (13.0%), VI (13.0%), and VII (15.6%), while level V showed involvement in 5.2% of patients. As comparing with ipsilateral neck, the risk of metastasis for contralateral neck tend to be lower (LMI: 16.4% vs. 5.2%). These results are in agreement with our previous study and the literature [13]. However, few studies have reported the outcomes of regional lymph node failure for locally advanced hypopharynx SCC after treatment with IMRT. Sommat et al. [14] reported a retrospective analysis of 58 patients (III–IV_b_ 95%) with hypopharyngeal cancer treated with curative intent RT. In Sommat’s study, 88% of patients managed to achieve complete response 3 months after completion of treatment, loco-regional recurrence remained the major cause of failure following curative intent RT. Most deaths occurred in patients who succumbed to loco-regional rather than systemic failure. However, only 50% of patients undergone IMRT in Sommat’s study, half part of patients treated using a 2-dimensional technique. Daly et al. [15] recruited 42 patients with newly diagnosed SCC of hypopharynx (23 patients) and larynx (19 patients) underwent IMRT, 11 postoperatively and 31 definitively at Stanford University Medical Center. Median follow-up was 30 months, 5 patients developed a loco-regional failure or had persistent disease, with a median time to failure of 12.1 months. Three local failures occurred within the high-dose region and 3 occurred in regional nodes. No marginal misses were observed. The author considered that loco-regional relapses occurred in the high-dose volumes, suggesting that target volume delineation was adequate but further dose-escalation and more aggressive treatment may be needed. Huang et al. [16] retrospectively reviewed 47 patients with locally advanced resectable SCC of hypopharynx underwent primary surgery or definitive IMRT with concurrent platinum-based chemotherapy (CCRT). The 5-year survival rate, disease-free survival, and loco-regional progression-free survival of surgery and CCRT group was 33 and 56%, 25 and 41%, 15 and 53%, respectively. Loco-regional progression was the main cause of failure in both groups. Eleven patients had neck failure; 8 in the ipsilateral neck, 2 in the contralateral neck, and 1 in the tracheostoma site. All were in-field failure in the PTV_2_ (60Gy). One retrospective study [17] reported by Chun et al. included 54 patients receiving definitive radiotherapy with or without chemotherapy. Thirty patients received IMRT and 24 patients received three dimensional conformal radiotherapy. With median follow-up time 42.3 months, there were 20 loco-regional failures discovered. Estimated crude loco-regional recurrence free survival at 3 years were 64.1%. Of the 20 loco-regional failures, 14 were isolated local failures, 4 were isolated regional nodal failures, and 2 were both. Of the 6 regional nodal failures, failures involved ipsilateral neck level II in 3 patients, ipsilateral neck level III in 1 patient, paraesophageal lymph node in 1 patient, and bilateral neck level II in 1 patient. Among the loco-regional failures, 17 were observed in the PTV high region, while 2 were in the PTV intermediate region and 1 patient had out-of-feld failure (paraesophageal lymph node), but was also accompanied by local failure within the PTV High region. Pignon et al. [18] found that IMRT failure in the low-neck supraclavicular field was very uncommon.

Our center has employed IMRT for the definitive treatment of head and neck cancers nearly for 10 years. Our study results demonstrated the poor outcome expected in hypopharyngeal cancer with median PFS rates were approximately 1 year after first-line treatment. The regional cervical lymph node recurrence and persistent disease remained the major cause of failure following curative intent of IMRT. Approximately 70% of nodal failures were observed in the PTV high or intermediate regions. In our study, the most commonly failure levels were the II (24.7%), and III (13.0%). However, the nodal failures at level IV, VIb and VII was uncommon, the rate of nodal failure only 1.3–2.6%. In our study, lymph node failure was mostly involved in ipsilateral neck, only 2 patients developed isolated level II failure in contralateral neck, and one patient developed level II failure in bilateral necks. Regarding our patients received IMRT enrolled in this study, more than half of patients have severe lymph node involvement and were not suitable candidates for selective lymph node dissection. Approximately 80% of them displayed lymph node metastasis with liquefactive necrosis in lymph nodes. After completion of IMRT treatment, majority of them in our cohort presented nodal residue. In our study, ENE with radiological evidence was observed significantly associated with lymph node recurrence and persistent diseases. In the recently released eighth edition of the AJCC TNM staging, ENE has been added as a prognostic variable for regional lymph node metastasis in addition to the number and size of metastatic lymph nodes [19]. Pitifully, because of extra capsular extension (for example vessels and soft tissue invasion), or nodal failures accompanied by local recurrence or distant metastasis, or severe late treatment toxicities, ultimately only 2 patients received a salvage node dissection within 6 months of follow-up time. Aside from 5 patients with local-regional failure received salvage surgery after definitive radiotherapy, most patients were received chemotherapy or combining with targeted therapy. Chun et al. [17] suggest that salvage surgery after definitive radiotherapy should be considered for patients who show residual disease after 6 months, because residual tumors show progression soon after 6 months.

In patients undergoing surgical resection with or without postoperative adjuvant IMRT. Seventeen patients were observed regional lymph node failure, 10 of them were isolated nodal failure, 4 patients accompanied by local recurrence, and 3 patients accompanied by distant metastasis (one patients occurred axillary lymphatic and scapula metastasis). Of the 16 patients with nodal failure, failures involved level II in 7 patients, levels III and VIb both in 4 patients, level VII in 5 patients. Furthermore, nodal failure involved in ipsilateral neck level IV and V was both one patient.

Regarding 46 patients undergone lymph node dissection with 35 ips- and 11 bilateral neck dissection in this study. Six of them observed contralateral neck failure, with level II in 4 patients, level III in 3 patients, level VIb and VII both in one patient. Among these 6 patients, 3 patients had received postoperative radiation with radiation dose of 50–66Gy. Previously multi-center randomized clinical trials have confirmed post-operative radiation or chemoradiation improves loco-regional control and overall survival in the presence of extra-capsular nodal extension [6, 7]. Although we fail to analyzed the correlation of pathologic ENE with node failure after surgery in our study, we found that the most commonly failure levels were the II (15.2%), III (8.7%), VIb (8.7%), and VII (10.9%). Comparing with patients receiving definitive radiotherapy, node failure rates at levels II and III were lower for patients receiving surgery as first-line treatment (15.2% vs. 24.7%; 8.7% vs. 13.0%), whereas, node failure at levels VIb and VII were exhibited higher (8.7% vs. 2.6%; 10.9% vs. 1.3%). The reason probably because the selective neck dissection always included the nodes in level II and III, whereas, the nodes in level VI and VII failed to remove from patients routinely in our study.

One retrospective study [13] include larynx (110 patients) and hypopharynx (26 patients) SCC undergoing total laryngectomy or pharyngolaryngectomy with neck dissection. Levels IIa and III were invaded in 28.7 and 25.7% of patients, respectively. Level VIb lymph-node involvement was 23.8% in patients who underwent level VIb neck dissection. Lymph-node recurrence rate was 10.3% in levels II to IV, and 13.2% in VIb. The author concluded that because high rate of involvement and recurrence of level VIb, systematic elective bilateral neck dissection might be needed. Previous retrospective studies [20, 21] indicated that pyriform sinus apex or postcricoid invasion, or tumor diameter exceeding 3.5 cm showed a trend in favor of paratracheal lymph node involvement. In our previous study, esophagus invasion was also highly correlated with increased risk of developing level VIb metastasis. It is noteworthy that lymph node at level VII (retropharyngeal lymph node) can not be removed routinely by surgery, and hardly be detected by imaging before surgery. Currently, there is no consensus regarding the delineation of lymphatic clinical target volume for post-operative radiation therapy for hypopharyngeal cancer. In present study, we found that not receiving postoperative radiation therapy was strongly associated with higher risk nodal failure. Five in 6 patients who failed to receiving postoperative radiation occurred nodal failure. Compared to the patients who received postoperative RT, the lymph node recurrence rate of level VII and VIb in ipsilateral neck was higher in patients who did not recevive postoperative RT (33.3% vs. 7.5%, *P* = 0.058; 33.3% vs. 5.0%, *P* = 0.022, Fig. 5). Furthermore, three patients (50.0%) occured nodal failure at level II in contralateral or bilateral necks for patients not receiving adjuvant radiation therapy, which was much higher than patients who recevive postoperative RT (50.0% vs. 2.5%, *P* < 0.001; OR = 0.026, 95%CI: 0.002–0.328). Based on results found in our study, irradiation of the level VIb and VII should be recommended, especially for the primary tumors originated from posterior pharyngeal wall (PPW), PPW invasion, postcricoid invasion, and esophagus invasion [22, 23].

The limitations of our study include its retrospective nature. The follow up time is relatively short. We did not perform the dosimetric analysis of the patterns of failure, and fail to confirm if CTV delineation is adequate. The prognosis associated factors, including the evaluation of the surgical margins, perineural invasion for hypopharyngeal cancer could not be taken into account.

## Conclusions

Based on our results, we concluded that whatever the treatment modality, levels II and III in ipsilateral neck were most commonly failure regions. The regional cervical lymph node recurrence and persistent disease remained the major cause of failure following curative intent of definitive IMRT. Because of high rate of node failure of level VIb and VII after surgery, post-operative radiation field should be include these territories, particularly in the setting of locally advanced disease. Our results provide a clear rationale for efforts in the future aimed at improving local-regional control, which including accurate target volume delineation, optimal prescribed radiation dose and fraction, possibly identification areas of radio-resistance within the tumour. Further clinical research is needed to assess the utilization of IMRT combined with novel systemic agents in locally advanced hypopharyngeal SCC.

## Data Availability

We declared that the materials and data of this study are available from the first author on reasonable request.

## Abbreviations

SCC: Squamous cell carcinoma
LMR: Lymphatic metastasis ratio
LMI: Lymphatic metastasis intensity

## References

[1] Fitzmaurice C, Allen C, Barber RM, Barregard L, Bhutta ZA, Brenner H, Global Burden of Disease Cancer Collaboration (2017). Global, Regional, and National Cancer Incidence, Mortality, Years of Life Lost, Years Lived With Disability, and Disability-Adjusted Life-years for 32 Cancer Groups, 1990 to 2015: A Systematic Analysis for the Global Burden of Disease Study. JAMA Oncol.

[2] Parkin DM, Bray F, Ferlay J, Pisani P (2005). Global cancer statistics, 2002. CA Cancer J Clin.

[3] Garneau JC, Bakst RL, Miles BA (2018). Hypopharyngeal cancer: a state of the art review. Oral Oncol.

[4] Chung EJ, Jeong WJ, Jung YH, Kwon SK, Kwon TK, Ahn SH, Sung MW, Keam B, Heo DS, Kim JH, Wu HG, Lee KW, Eom KY, Rho YS (2019). Long-term oncological and functional outcomes of induction chemotherapy followed by (chemo) radiotherapy vs definitive chemoradiotherapy vs surgery-based therapy in locally advanced stage III/IV hypopharyngeal cancer: multicenter review of 266 cases. Oral Oncol.

[5] Lefebvre JL, Andry G, Chevalier D, Luboinski B, Collette L, Traissac L, de Raucourt D, Langendijk JA (2012). EORTC head and neck Cancer group. Laryngeal preservation with induction chemotherapy for hypopharyngeal squamous cell carcinoma: 10-year results of EORTC trial 24891. Ann Oncol.

[6] Argiris A, Karamouzis MV, Raben D, Ferris RL (2008). Head and neck cancer. Lancet.

[7] Pfister DG, Spencer S, Adelstein D (2018). NCCN Clinical Practice Guidelines in Oncology (NCCN Guidelines) Head and Neck Cancer Version 1.

[8] Biau J, Lapeyre M, Troussier I, Budach W, Giralt J, Grau C, Kazmierska J, Langendijk JA, Ozsahin M, O'Sullivan B, Bourhis J, Grégoire V (2019). Selection of lymph node target volumes for definitive head and neck radiation therapy: a 2019 update. Radiother Oncol.

[9] Grégoire V, Ang K, Budach W, Grau C, Hamoir M, Langendijk JA, Lee A, Le QT, Maingon P, Nutting C, O'Sullivan B, Porceddu SV, Lengele B (2014). Delineation of the neck node levels for head and neck tumors: a 2013 update. DAHANCA, EORTC, HKNPCSG, NCIC CTG, NCRI, RTOG, TROG consensus guidelines. Radiother Oncol.

[10] Forastiere AA, Zhang Q, Weber RS, Maor MH, Goepfert H, Pajak TF (2013). Long-term results of RTOG 91-11: a comparison of three nonsurgical treatment strategies to preserve the larynx in patients with locally advanced larynx cancer. J Clin Oncol.

[11] Forastiere AA, Goepfert H, Maor M, Pajak TF, Weber R, Morrison W (2003). Concurrent chemotherapy and radiotherapy for organ preservation in advanced laryngeal cancer. N Engl J Med.

[12] Rabuzzi DD, Chung CT, Sagerman RH (1980). Prophylactic neck irradiation. Arch Otolaryngol.

[13] Rivière D, Mancini J, Santini L, Giovanni A, Dessi P, Fakhry N (2018). Lymph-node metastasis following total laryngectomy and total pharyngolaryngectomy for laryngeal and hypopharyngeal squamous cell carcinoma: frequency, distribution and risk factors. Eur Ann Otorhinolaryngol Head Neck Dis.

[14] Sommat K, Yong SK, Fong KW, Tan TW, Wee JT, Soong YL (2017). A 13-year single institutional experience with definitive radiotherapy in Hypopharyngeal Cancer. Ann Acad Med Singap.

[15] Daly ME, Le QT, Jain AK, Maxim PG, Hsu A, Loo BW, Kaplan MJ, Fischbein NJ, Colevas AD, Pinto H, Chang DT (2011). Intensity-modulated radiotherapy for locally advanced cancers of the larynx and hypopharynx. Head Neck.

[16] Huang WY, Jen YM, Chen CM, Su YF, Lin CS, Lin YS, Chang YN, Chao HL, Lin KT, Chang LP (2010). Intensity modulated radiotherapy with concurrent chemotherapy for larynx preservation of advanced resectable hypopharyngeal cancer. Radiat Oncol.

[17] Chun SJ, Keam B, Heo DS, Kim KH, Sung MW, Chung EJ, Kim JH, Jung KC, Kim JH, Wu HG (2018). Optimal timing for salvage surgery after definitive radiotherapy in hypopharyngeal cancer. Radiat Oncol J.

[18] Pignon JP, le Maitre A, Maillard E, Bourhis J, Group M-NC (2009). Meta-analysis of chemotherapy in head and neck cancer (MACH-NC): an update on 93 randomised trials and 17,346 patients. Radiother Oncol.

[19] Lydiatt WM, Patel SG, O'Sullivan B (2017). Head and neck cancers-major changes in the American joint committee on cancer eighth edition cancer staging manual. CA Cancer J Clin.

[20] Chung EJ, Kim GW, Cho BK, Park HS, Rho YS (2016). Pattern of lymph node metastasis in hypopharyngeal squamous cell carcinoma and indications for level VI lymph node dissection. Head Neck.

[21] Dequanter D, Shahla M, Zouaoui Boudjeltia K, Paulus P, Lothaire P (2013). Neck and mediastinal node dissection in pharyngolaryngeal tumors. Eur Ann Otorhinolaryngol Head Neck Dis.

[22] Harada R, Isobe K, Watanabe M, Kobayashi H, Horikoshi T, Motoori K (2012). The incidence and significance of retropharyngeal lymph node metastases in hypopharyngeal cancer. Jpn J Clin Oncol.

[23] Wu Z, Deng XY, Zeng RF, Su Y, Gu MF, Zhang Y (2013). Analysis of risk factors for retropharyngeal lymph node metastasis in carcinoma of the hypopharynx. Head Neck.

